# Intratympanic steroids injection is effective for the treatment of drop attacks with Ménière's disease and delayed endolymphatic hydrops: A retrospective study

**DOI:** 10.1097/MD.0000000000005767

**Published:** 2016-12-30

**Authors:** Bo Liu, Yangming Leng, Renhong Zhou, Jingjing Liu, Dongdong Liu, Su-Lin Zhang, Wei-Jia Kong

**Affiliations:** Department of Otolaryngology, Union Hospital, Tongji Medical College, Huazhong University of Science and Technology, Wuhan, Hubei Province, PR China.

**Keywords:** dexamethasone, endolymphatic hydrops, gentamicin, injection, inner ear

## Abstract

Drop attack (DA) associated with Ménière's disease (MD) and delayed endolymphatic hydrops (DEH) is not common and may cause life-threatening clinical problems. The intratympanic dexamethasone (ITD) is one of primary treatments for MD or DEH. Our study investigated the effect of ITD on the DA associated with endolymphatic hydrops (EH).

We retrospectively reviewed 10 patients with MD- and DEH-associated DA between January 2009 and December 2013 in Outpatient Department of Otolaryngology, Union Hospital, Wuhan, China. Among them, 7 patients (5 cases with MD, 2 cases of DEH) received ITD (4 times, on weekly basis). Further repeated ITD courses or intratympanic gentamicin (ITG) were given if the vertigo was not satisfactorily controlled. The number of DA and status of vertigo control after intratympanic injection were evaluated. After a follow-up study lasting from 19 to 35 months, DA in 5 cases (71.4%) disappeared after initial ITD course. In 2 cases, DA was altogether controlled after an additional intratympanic injection (repeated ITD or/and ITG).

This study showed that ITD promises to be a first-line conservative treatment for MD- or DEH-related DA since the steroid possesses no inner-ear toxicity. Furthermore, for MD- or DEH-related DA refractory to ITD, ITG can be an effective alternative.

## 1 Introduction

Clinically, Ménière's Disease (MD) and delayed endolymphatic hydrops (DEH) share some manifestations, including episodic vertigo attacks, sensorineural hearing loss, tinnitus, or ear pressure.^[1,2]^ Histopathologically, both MD and DEH have endolymphatic hydrops.^[3]^ In 1936, Tumarkin,^[4]^ for the first time, described the symptom of sudden drop attack (DA) in patients with MD, speculating that falling reaction results from a mechanical deformation of otolith organs, and named it an “otolithic catastrophe.” Moreover, DEH patients may also have the symptoms of DA.^[5]^ Now, it is believed that the DA, characterized by a sudden spontaneous fall, without loss of consciousness, is caused by an abruptly reduced muscle tone of lower limbs.^[6]^ DA is a symptom, not a disease entity. Etiologically, its’ causes might involve heart, brain, muscles, and might even be of psychological origin.^[7]^ It was found to be associated with vertigo disorders such as superior canal dehiscence,^[8]^ migraine,^[6]^ and other peripheral vestibulopathies.^[5]^ Of note, in 1960, Sheldon^[9]^ reported that drop accounted for about 1/4 of 500 consecutive falls in older patients. Therefore, DA associated with MD and DEH represents a life-threatening clinical problem, which needs to be addressed seriously.^[10]^

Essentially, treatment for MD and DEH is to control vertigo and preserve existing hearing. Patients with MD and DEH can be treated with a variety of therapies, including lifestyle changes, medications, intratympanic injections,^[11]^ Meniett device,^[12]^ and surgical interventions.^[1]^ Clinically, MD- and DEH-associated DA might disappear spontaneously in some patients.^[13,14]^ On the other hand, a study by Perez-Fernandez et al^[15]^ exhibited that patients who experienced Tumarkin attacks were more disabled and they suggested that treatment should be carefully planned to be as conservative as possible in terms of hearing impairment. Additionally, in some patients, the DA is either very serious or intractable that the aggressive treatment is warranted,^[13]^ including labyrinthectomy, vestibular neurectomy, and intratympanic gentamicin injection (ITG).^[10]^ In the past decades, the intratympanic dexamethasone (ITD), a nondestructive treatment, has become one of primary treatments for MD or DEH.^[11]^ In our clinic, ITD was found to be effective for vertigo control in some MD or DEH patients with DA. To our knowledge, until now, few studies focused on the effect of ITD on the DA associated with MD or DEH. In this study, we retrospectively investigated the efficacy of ITD for the treatment of MD- or DEH-associated DA.

## 2 Patients and methods

### 2.1 Subjects

We reviewed medical records of all MD and DEH patients who had experienced DA in our hospital. From January 2009 to December 2013, 150 consecutive patients with MD and 14 patients with DEH visited our Outpatient Department of Otolaryngology, Union Hospital, Wuhan, China. The diagnosis of MD was based on the criteria proposed by the Committee on Hearing and Equilibrium of the American Academy of Otolaryngology Head and Neck Surgery (AAO-HNS) in 1995.^[16]^ The diagnosis of DEH was established against previously published criteria.^[11]^ Among these subjects, 10 patients with MD- and DEH-associated DA were included according to following criteria: (1) definite Meniere's disease and delayed endolymphatic hydrops, (2) sudden drop attacks without warning signs, (3) no loss of consciousness, (4) other causes eliminated, including cardiac and neurological diseases, and (5) no musculoskeletal conditions that interfere with postural stability.

In addition, criteria of exclusion included: (1) having undergone the intratympanic injection or surgery, (2) having other concomitant vertigo disorders, such as vestibular migraine, benign paroxysmal positional vertigo, and central vertigo.

This study was conducted in accordance with the tenets of the Declaration of Helsinki and was approved by the Ethical Committee of Union Hospital affiliated to Tongji Medical College of Huazhong University of Science and Technology, Wuhan, China. Informed consent was obtained from each patient.

### 2.2 Pretreatment evaluations

Before diagnosis, detailed history had been recorded for each subject. All 10 patients were subjected to comprehensive neurotological tests for evaluating the audio-vestibular function, including pure tone audiometry, acoustic immitance measurement, electrocochleogram (ECochG), caloric test, and sensory organization test (SOT) battery of the computerized dynamic posturography (CDP) (NeuroCom International, Portland, OR). For ECochG, an enhanced summating potential (SP)/action potential (AP) ratio (≥0.4) was considered to be positive. In the caloric test with vedionystagmugraphy, the unilateral weakness (UW) was defined according to the conventional Jongkees formula.^[11]^ The Dix-Hallpike and Roll test were also vedio nystagmu graphically conducted. With the CDP test, the composite score of SOTs was calculated and evaluated according to the manufacturer's criteria.

Magnetic resonance imaging scan on the internal auditory canal and cerebellopontine angle was performed in all subjects to exclude tumors or other space-occupying lesions.

### 2.3 Intratympanic injection procedure

All patients were treated initially by change of lifestyle and medications, including betahistine, diuretics, or vasodilators for at least 6 months. Among the 10 patients, 3 did not receive ITD because vertigo and DAs were controlled or alleviated after these conservative therapies. The ITD was administered to the other 7 patients who failed to response to the conservative treatments.

ITD was administered in an office setting. The local anesthesia and intratympanic injection were performed by using previously reported techniques.^[11]^ The intratympanic dexamethasone injection (ITD), as a fixed protocol, was given once a week for 4 consecutive weeks, constituting 1 ITD course. After initial ITD course, the patients were observed for Meniere's attacks for 6 months. If vertigo attack was not effectively controlled, another ITD course (repeated ITD) was given. During another 6 months after the second ITD, vertigo control was evaluated. If patients were not satisfied with the effect of vertigo treatment, ITG with modified titration protocol would be given.^[17]^

### 2.4 Follow-up and evaluation

All patients receiving the initial ITD course were followed up or contacted by telephone by the time this paper was prepared. The numbers of DA before and after intratympanic injection were recorded. The vertigo score was calculated on a 6-point scale (Class A-F) according to the guidelines of AAO-HNS(1995).^[16]^

## 3 Results

In this retrospective study, 10 patients with MD- and DEH-related DA were identified. Clinical information and findings of audio-vestibular evaluation are listed in Table 1. According to the diagnostic criteria, 8 patients were found to have unilateral definite MD and 2 had ipsilateral DEH. Of 8 cases of MD, 2 were males and 6 females, with their age ranging from 31 to 62 years (mean = 51 years). In the remaining 2 DEH patients, one was male and the other was female, with their age being 41 and 25 years, respectively.

**Table 1:**
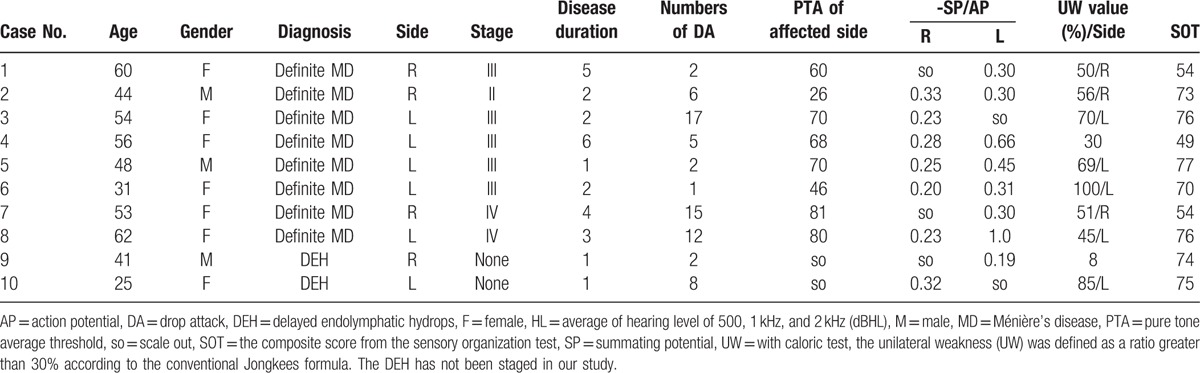
Pretreatment evaluations of audio-vestibular function in 10 cases with drop attacks secondary to Ménière's disease and delayed endolymphatic hydrops.

### 3.1 Clinical assessments

Among the 8 cases of MD-related DA, 1 was at stage II, 5 stage III, and 2 stage IV, according to the AAO-HNS guidelines.^[16]^ The left side was affected in 5 cases and the right side was involved in 3 patients. The disease duration lasted for 3.1 years on average (ranging from 1 to 6 years). The number of DA before the diagnosis ranged from 1 to 17 (mean number = 7.5 times). The mean pure tone average threshold of the affected side was 62.6 dB (ranging from 26 to 81 dB). In 8 patients, the ratio of –SP/AP was abnormal in 6 patients (75%), and normal in 2 cases (25%). Of 8 patients with MD-related DA, 7 cases (87.5%) showed UW in the affected ear. Three patients had abnormal composite scores of SOTs (37.5%).

Of 2 patients with ipsilateral DEH, the number of DA before the diagnosis was 2 and 8 time(s), respectively. The profound sensorineural hearing loss was found on the right (case 9) and left side (case 10). Abnormal ratios of –SP/AP were recorded in these 2 DEH patients. One patient had UW (case 10). All the 2 patients with ipsilateral DEH had normal composite scores of SOTs.

All subjects responded normally in the gaze test, saccades test, smooth pursuit test, and optokineticnystagmus test. Dix-Hallpike and Roll tests revealed no positional vertigo and nystagmus.

### 3.2 Efficacy of intratympanic injection on DA and vertigo control

Effects of ITD on the DA and vertigo are presented in Table 2. In 7 patients treated by the intratympanic infusion, the DA disappeared after initial ITD course in 5 cases (71.4%). Of 7 patients receiving the initial ITD, 5 received only 1 course of intratympanic injection. Complete vertigo control (Class A) was achieved in 2 patients (cases 2 and 8), and substantial control (Class B) was accomplished in 3 cases (cases 4, 9, and 10).

**Table 2:**
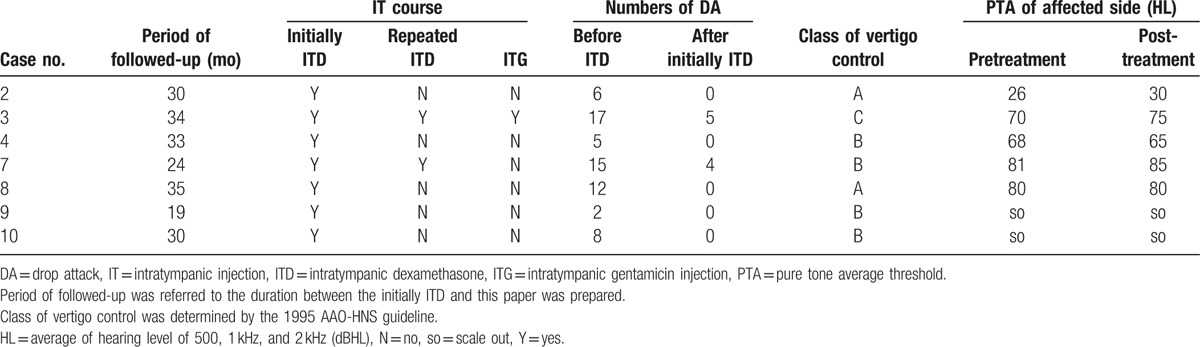
Outcomes of intratympanic injections for patients with drop attacks secondary to Ménière's disease and delayed endolymphatic hydrops.

In case 7, the number of DA was reduced after initial ITD course (from 15 times to 4 times), and DA disappeared after a repeated ITD course. This case still suffered from vertigo attacks after an initial ITD course. Over a 6-month period, this patient received a repeated ITD course, and substantial control (Class B) was achieved.

After 1 course of ITD, 1 patient (case 3) still had DA, but a 6-month follow-up showed that the number of DA dropped and the number of vertigo attacks was reduced significantly. Then, since the vertigo attack was not satisfactorily relieved (Class C), this patient underwent a repeated ITD course and, after another 6 months, 2 intratympanic gentamicin injections. After ITG, DA disappeared altogether and vertigo was substantially controlled (Class B).

Following ITD or ITG, no patients showed conspicuous hearing loss and no patients reported any complications such as otitis media and tympanic membrane perforation during the followed-up period.

## 4 Discussion

In this study, we employed ITD to treat 7 patients with MD- and DHE-related DA and DA disappeared after initial ITD course in 5 cases, with a success rate of 71.4%. Over past years, many treatments have been used for the treatment of MD- or DEH-related DA. Black et al^[7]^ performed several types of surgeries to treat MD-associated DA. ITG, as a destructive treatment, could achieve success rates ranging from 60% to 100% in controlling MD-related DA.^[10,18]^ Since both ITG and ITD are well-established treatment for MD, we are led to speculate that ITD may also be used for controlling MD- or DEH-related DA. ITD, free of inner ear toxicity, may attain a compromise between vertigo control and hearing loss. In this study, 1 course of ITD achieved a success rate of 71.4% (5/7), in terms of control of MD- and DEH-related DA, and the initial plus an additional ITD course registered a success rate of 85.7% (6/7). In 1 patient (case 3) in our series, DA disappeared completely after 2 courses of ITD followed by an ITG injection. In fact, our results and previous findings suggested that, ITG, as a more long-lasting and effective alternative, can serve as a subsequent treatment if repeated ITD fails to effectively relieve MD-related DA.^[10,19]^ Although the DA control rate of ITD was not as high as that of ITG, ITD, as a conservative and nondestructive procedure, can not only effectively control DA but also avoid post-ITG hearing loss and disequilibrium associated with use of gentamicin.

Although the present study showed an encouraging result, the exact mechanism of ITD working on the DA is not fully understood. In patients with MD and DEH, DA might result from an abrupt mechanical deformation of the otolithic membrane due to an increased pressure gradient within the inner ear, which leads to a reflex-like vestibulospinal loss of postural tone.^[14]^ A significantly higher incidence of otolithic membrane injury was pathologically proven in patients with MD- and DEH-related DA than in their counterparts without EH.^[20]^ Therefore, we speculate that ITD might work on the MD- or DEH-related DA by lowering the elevated pressure gradients within the inner ear, and subsequently ameliorating the mechanical deformation of otolithic membranes. It is well-known that the endolymphatic hydrops bears relation with an altered water transport, such as increased endolymph secretion and/or decreased endolymph re-absorption. Aquaporins (AQPs) are a family of water channel proteins, allowing trans-membrane water transport, and are ubiquitously found in different cell types of the inner ear. A recent study detected the presence of AQP3 in human utricle and found that dexamethasone could promote water re-absorption by upregulating AQP3 expression.^[21]^ On the basis of the findings, we are led to speculate that effect of ITD on MD- and DEH-related DA might be explained by the fact that dexamethasone promotes endolymphatic water re-absorption.

Our results also showed that DA, in 3 MD patients, disappeared when the vertigo was controlled or alleviated after conservative therapies. According to Janzen and Russell^[22]^ and Baloh *et al*,^[14]^ some MD-related DA had spontaneous remission. According to previous studies, the natural course of MD, in terms of vertigo, varied with different individuals. Recent studies demonstrated that the rate of spontaneous remission was relatively lower in MD. In a longitudinal study, Perez-Garrigues et al^[23]^ found that only 37.7% of patients receiving medical treatment experienced complete remission of vertigo within the first 2 years. A 2-year follow-up study reported a complete remission rate of 8% in nonoperated patients receiving medical treatment.^[24]^ Our study demonstrated, in some MD patients, both vertigo and DA symptoms were ameliorated after medical treatment, suggesting that these patients can be treated with conservative treatment.

DA was not a common clinical manifestation in patients with MD or DEH and the sample size of the study was relatively small. Large-sized controlled studies and long-term follow-up surveys are warranted to understand the effect of intratympanic steroid on DA associated with endolymphatic hydrops.

## 5 Conclusions

This study showed that ITD promises to be a first-line conservative treatment for MD- or DEH-related DA since the steroid possesses no inner-ear toxicity. Furthermore, for MD- or DEH-related DA refractory to ITD, ITG can be an effective alternative.

## References

[1] Sajjadi H Paparella MM Meniere's disease. Lancet 2008;372:406–14. 18675691 10.1016/S0140-6736(08)61161-7

[2] Kamei T Delayed endolymphatic hydrops as a clinical entity. Int Tinnitus J 2004;10:137–43. 15732511

[3] Schuknecht HF Suzuka Y Zimmermann C Delayed endolymphatic hydrops and its relationship to Meniere's disease. Ann Otol Rhinol Laryngol 1990;99:843–53. 2241008 10.1177/000348949009901101

[4] Tumarkin A The otolithic catastrophe: a new syndrome. Brit Med J 1936;2:175–7. 20779991 10.1136/bmj.2.3942.175 PMC2457095

[5] Lee H Yi HA Lee SR Drop attacks in elderly patients secondary to otologic causes with Meniere's syndrome or non-Meniere peripheral vestibulopathy. J Neurol Sci 2005;232:71–6. 15850585 10.1016/j.jns.2005.01.012

[6] Ishiyama G Ishiyama A Baloh RW Drop attacks and vertigo secondary to a non-Meniere otologic cause. Arch Neurol 2003;60:71–5. 12533091 10.1001/archneur.60.1.71

[7] Black FO Effron MZ Burns DS Diagnosis and management of drop attacks of vestibular origin: Tumarkin's otolithic crisis. Otolaryngol Head Neck Surg 1982;90:256–62. 6810273 10.1177/019459988209000221

[8] Brantberg K Ishiyama A Baloh RW Drop attacks secondary to superior canal dehiscence syndrome. Neurology 2005;64:2126–8. 15985585 10.1212/01.WNL.0000165953.48914.B0

[9] Sheldon JH On the natural history of falls in old age. Brit Med J 1960;2:1685–90. 20789006 10.1136/bmj.2.5214.1685 PMC2098310

[10] Viana LM Bahmad F Jr Rauch SD Intratympanic gentamicin as a treatment for drop attacks in patients with Meniere's disease. Laryngoscope 2014;124:2151–4. 24729095 10.1002/lary.24716

[11] Liu B Zhang S Leng Y Intratympanic injection in delayed endolymphatic hydrops. Acta Otolaryngol 2015;135:1016–21. 26050741 10.3109/00016489.2015.1052984

[12] Huang W Liu F Gao B Clinical long-term effects of Meniett pulse generator for Meniere's disease. Acta Otolaryngol 2009;129:819–25. 18923942 10.1080/00016480802468146

[13] Dallan I Bruschini L Nacci A Drop attacks and vertical vertigo after transtympanic gentamicin: diagnosis and management. Acta Otorhinolaryngol Italica 2005;25:370–3. PMC2639899 16749606

[14] Baloh RW Jacobson K Winder T Drop attacks with Meniere's syndrome. Ann Neurol 1990;28:384–7. 2241120 10.1002/ana.410280314

[15] Perez-Fernandez N Montes-Jovellar L Cervera-Paz J Auditory and vestibular assessment of patients with Meniere's disease who suffer Tumarkin attacks. Audiol Neurootol 2010;15:399–406. 20389064 10.1159/000310899

[16] Committee on Hearing and Equilibrium guidelines for the diagnosis and evaluation of therapy in Menière's disease. American Academy of Otolaryngology—Head and Neck Foundation, Inc. Otolaryngol Head Neck Surg 1995;113:181–5. 7675476 10.1016/S0194-5998(95)70102-8

[17] Liu B Leng YM Shi H Modified titration intratympanic gentamicin injection for unilateral intractable Meniere's disease. J Huazhong Univ Sci Technolog Med Sci 2015;35:747–51. 26489633 10.1007/s11596-015-1501-7

[18] Odkvist LM Bergenius J Drop attacks in Meniere's disease. Acta Otolaryngol Suppl 1988;455:82–5. 3265265 10.3109/00016488809125064

[19] Leng Y Liu B Zhou R Repeated courses of intratympanic dexamethasone injection are effective for intractable Meniere's disease. Acta Otolaryngol 2016;1–7. 10.1080/00016489.2016.1224920 27650470

[20] Calzada AP Lopez IA Ishiyama G Otolithic membrane damage in patients with endolymphatic hydrops and drop attacks. Otol Neurotol 2012;33:1593–8. 23064391 10.1097/MAO.0b013e318271c48b PMC5358090

[21] Nevoux J Viengchareun S Lema I Glucocorticoids stimulate endolymphatic water reabsorption in inner ear through aquaporin 3 regulation. Pflugers Arch 2015;467:1931–43. 25339224 10.1007/s00424-014-1629-5

[22] Janzen VD Russell RD Conservative management of Tumarkin's otolithic crisis. J Otolaryngol 1988;17:359–61. 3230608

[23] Perez-Garrigues H Lopez-Escamez JA Perez P Time course of episodes of definitive vertigo in Meniere's disease. Arch Otolaryngol Head Neck Surg 2008;134:1149–54. 19015442 10.1001/archotol.134.11.1149

[24] Kitahara T Kubo T Okumura S Effects of endolymphatic sac drainage with steroids for intractable Meniere's disease: a long-term follow-up and randomized controlled study. Laryngoscope 2008;118:854–61. 18520184 10.1097/MLG.0b013e3181651c4a

